# Identification of disease comorbidity through hidden molecular mechanisms

**DOI:** 10.1038/srep39433

**Published:** 2016-12-19

**Authors:** Younhee Ko, Minah Cho, Jin-Sung Lee, Jaebum Kim

**Affiliations:** 1 Department of Clinical Genetics, Department of Pediatrics, Yonsei University College of Medicine, Seoul 03722, South Korea; 2 Department of Stem Cell and Regenerative Biology, Konkuk University, Seoul 05029, South Korea

## Abstract

Despite multiple diseases co-occur, their underlying common molecular mechanisms remain elusive. Identification of comorbid diseases by considering the interactions between molecular components is a key to understand the underlying disease mechanisms. Here, we developed a novel approach utilizing both common disease-causing genes and underlying molecular pathways to identify comorbid diseases. Our approach enables the analysis of common pathologies shared by comorbid diseases through molecular interaction networks. We found that the integration of direct genetic sharing and indirect high-level molecular associations revealed significantly strong consistency with known comorbid diseases. In addition, neoplasm-related diseases showed high comorbidity patterns within themselves as well as with other diseases, indicating severe complications. This study demonstrated that molecular pathway information could be used to discover disease comorbidity and hidden biological mechanism to understand pathogenesis and provide new insight on disease pathology.

The core in comorbidity research lies in the elucidation of pathological properties of diseases and their coordinated activities at molecular level. In recent years, remarkable advances in the understanding of human disease mechanisms have provided increasing evidence that most complex diseases are caused by the breakdown of concerted activities of many genes involved in common or related cellular processes^1^,^2^,^3^. The coexistence of two or more diseases in an individual raises the question about their underlying common etiological pathways. The study of comorbidity patterns of diseases could help us understand the underlying molecular disease mechanisms and identify potential novel disease-causing genes or associated biological pathways^4^.

Several studies have investigated comorbidity patterns of diseases^5^,^6^,^7^,^8^,^9^,^10^ by considering several biological factors relevant to existing comorbidities. The etiology of comorbid diseases occurring in an individual can be explained by two mechanisms. First, directly shared biological factors such as common disease genes can cause comorbid diseases. Second, comorbid diseases can occur together since they are co-regulated by high-level biological mechanisms such as the same cellular pathways. Most existing studies have focused on the first mechanism. For example, direct overlap of disease-associated genes has been identified as a one of critical factors to explain the comorbid diseases^11^. The number of direct protein-protein interactions (PPIs) between causative proteins of two diseases has also been considered to explain the hidden comorbidity patterns^7^,^12^,^13^. Recently, symptom similarity has been used to explain the unexpected association among diseases, disease etiology, and drug design^9^. However, comorbid diseases are more likely to co-occur because disease-associated genes are indirectly co-regulated by underlying common biological mechanisms^4^,^8^,^14^. In order to study the pathology of comorbid diseases, both direct sharing of disease-associated genes or PPIs and indirect common mechanisms should be considered.

In this study, we combined functional relations between protein coding genes and biological modules associated with them to investigate the etiology of unexplained comorbidity and to elucidate the molecular origins or underlying mechanisms of such comorbid diseases. First, we compiled large-scale gene-disease associations by integrating four well-known disease databases. We then developed a method to identify comorbid diseases through functional association networks. For clinical validation, the US Medicare database^15^ was used. These analyses discovered the associated disease mechanisms underlying comorbid diseases and highly co-emerged clinical disease categories. We used biological pathways interleaved within indirect relations between disease-associated genes to explore novel comorbidity patterns in a systematic way where those genes were linked at the higher molecular network-level. Integration of disease genes and molecular interactions under functional networks enabled us to investigate unknown co-occurred disease pairs and their pathobiological properties.

## Results

### Integration of disease databases and extraction of disease-gene associations

**Figure 1:**
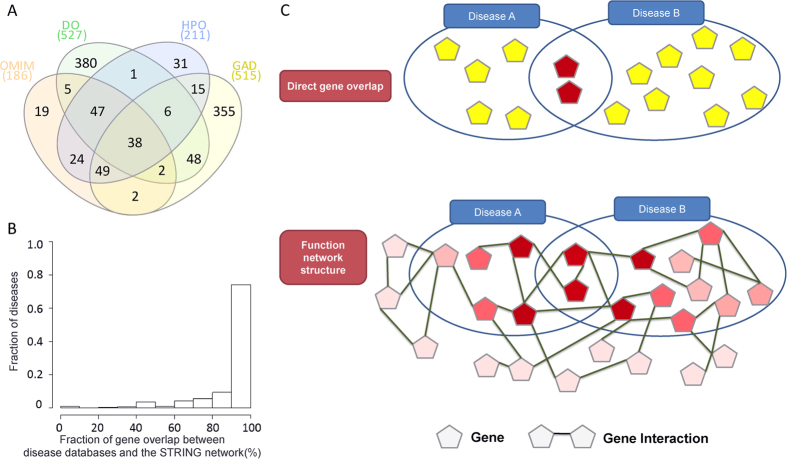
Statistics of four integrated disease databases (i.e., OMIM, DO, HPO, and GAD) and the overall schema of three representative quantities to identify disease comorbidity. (**A**) Disease overlap among four disease databases. The number in parentheses represents the total number of genes in each database. (**B**) Disease gene coverage of the integrated disease database in comparison with STRING network. The x-axis represents the proportion of overlap between associated genes of a disease and all genes in the STRING network. The y-axis indicates the fraction of diseases. The fraction of diseases (more than 80% of disease genes are covered by STRING) is over 95%. (**C**) Two different strategies to represent the degree of comorbidity between diseases A and B. “Direct gene overlap” and “Function network structure” are used to consider overlap between associated genes of the two diseases and the number of direct as well as indirect interactions between associated genes of the two diseases in a function network, respectively. The “Function network structure” strategy to explain the disease comorbidity utilizes disease-associated genes as well as the neighborhood genes which are connected to the disease-associated genes. In our study, the STRING interaction database has been used to identify the functional interactions.

To cover extensive disease-gene associations, four well-known disease databases were integrated (see Methods), which dramatically increased disease coverage (Fig. 1A). Since different disease databases were collected based on different biological evidences, coverages of these databases were very different from each other. Our integrated database covered 897 diseases (increase of 17.56% compared with previous study^12^) overlapped with Medicare data. Most (79.62%) genes were also covered by STRING network^16^ used for network-based comorbidity inference (Fig. 1B).

### Identification of comorbid diseases

To identify comorbidity patterns of diseases, two quantities (direct sharing of disease-associated genes and the commonality of protein-protein interactions (PPIs) between candidate comorbid diseases (Fig. 1C)) have been studied^7^,^11^,^12^,^15^,^17^. These studies have shown some degree of correlation with the actual comorbidity patterns of diseases obtained from clinical data. However, comorbid diseases co-occur not only because they share genes or protein interactions, but also because their pathobiological properties involve a whole cascade of common perturbed cellular mechanisms. Therefore, only considering these two quantities would not be enough to explain comorbidity patterns and their pathobiological properties.

To address this issue, we developed a new approach by considering high-level molecular associations such as biological pathways in addition to the two traditional quantities (Fig. 1C). Recently, EnrichNet (network-based gene set enrichment analysis) has been introduced for a new gene set enrichment analysis by considering a gene interaction network^18^. We adapted similar ideas of EnrichNet to infer the degree of comorbidity between two diseases that reflect functional closeness among a group of disease-associated genes as well as associated biological mechanisms. Specifically, given a set of genes and a gene interaction network, the level of reachability (called the XD score) from one gene set to the other gene set could be quantified by random walk with restart algorithm (Methods). Although different diseases can co-occur due to various reasons including common symptoms, shared molecular mechanisms, shared genes, or drug effects, recent comorbidity studies are only applicable for disease pairs that directly share molecular components (e.g. common disease genes or common PPIs). This may lead to the missing of a large number of meaningful comorbidity patterns, which can result in incomplete understanding of pathobiological properties of diseases. Therefore, our approach will be particularly useful to identify hidden comorbidity patterns in cases where diseases share no common gene in the same molecular or cellular processes.

### Evaluation through real patient data

Our results were evaluated with the US Medicare data. We explained the etiology of comorbid diseases with their common molecular mechanisms, which is represented as the XD score of two diseases. Thus, we calculated the XD scores of all disease pairs, and measured the amount of correlation with the following two traditional scores for disease comorbidity: relative risk (RR) and phi-correlation (PHI). For each disease pair, both RR and PHI are calculated based on the amount of patients with common diseases from the US Medicare data (see Methods)^7^,^12^,^15^,^19^. Although RR and PHI describe how often two diseases actually co-occur in clinical data, both measures have their own intrinsic biases. Therefore, we used both scores to quantify the comorbidity. To determine the effectiveness of our approach, the correlation between RR/PHI and the following two quantities or their combinations were compared: (i) the number of common genes between two diseases (NG), (ii) the XD score calculated by our approach (Supplementary Table 1).

**Figure 2:**
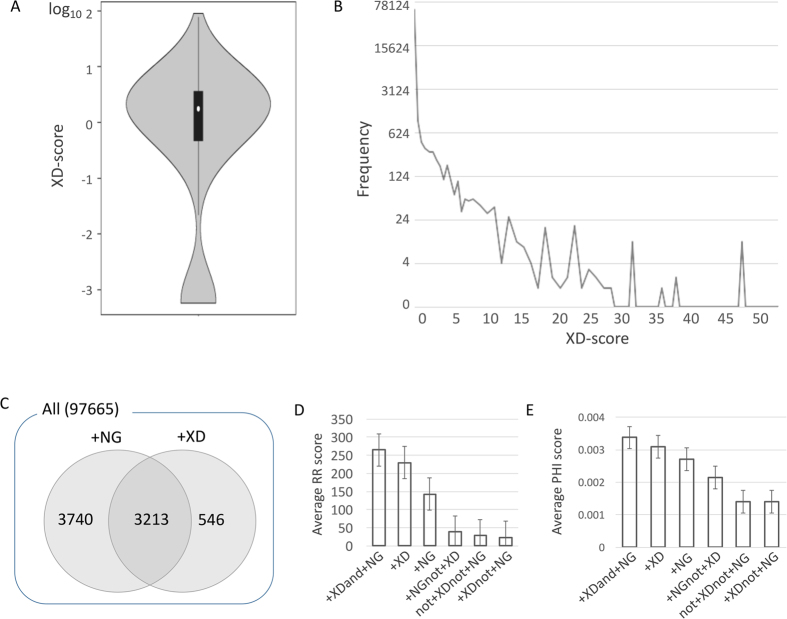
Statistics of comorbidity measures for disease pairs in the US Medicare data. (**A**) The distribution of the log-scaled XD scores. (**B**) The distribution of the positive XD scores. (**C**) The numbers of disease pairs chosen by different quantities (+NG: disease pairs having at least one common gene, +XD: disease pairs having the positive XD scores). (**D**) The average and standard errors of RR scores of disease pairs chosen by different quantities. (**E**) The average and standard errors of PHI scores of disease pairs chosen by different quantities. (+XDand + NG: disease pairs having both the positive XD scores and at least one common gene, +NGnot + XD: disease pairs having at least one common gene but without the positive XD scores, +XDnot + NG: disease pairs having the positive XD scores but without sharing genes, and not + XDnot + NG: disease pairs having the negative or zero XD scores without sharing any gene).

The overall distribution of the XD score of all disease pairs is shown in Fig. 2 (Fig. 2A, all XD scores; Fig 2B, only positive XD scores). To examine the effectiveness of our approach more deeply, we compared the distribution of RR and PHI scores of disease pairs extracted from the following different criteria: disease pairs with at least one common gene (+NG), with the positive XD score (+XD), with both the positive XD score and at least one common gene (+XDand + NG), with at least one common gene but without the positive XD score (+NGnot + XD), with the positive XD score but without sharing genes (+XDnot + NG), and with the negative or zero XD scores without common genes (not + XDnot + NG). In this classification, +NG (at least one common gene) and +XD (positive XD score) were used as cutoffs because it has been shown that disease pairs having shared genes (e.g. NG > 0) have high comorbidity^7^,^8^,^12^,^15^, and the negative XD score represents that the comorbidity level of two disease pairs is low and those set of disease genes have less than average connections (Methods). Among a total of 97,665 disease pairs, the number of disease pairs having at least one overlapped disease-associated genes and disease pairs having the positive XD score were 6,953 (7%) and 3,759 (4%), respectively. A total of 3,213 (3%) disease pairs were common (Fig. 2C). As shown in Fig. 2D and E, disease pairs selected by both the XD score and NG (i.e. the +XDand+NG category) revealed the highest comorbidity patterns (i.e., the highest average RR and PHI scores) compared to disease pairs in other categories. In addition, disease pairs without +XDor+NG showed relatively very low comorbidity patterns. There was no big difference between results using RR and PHI in terms of such correlation. This demonstrated that the identification of comorbid diseases could be more accurately achieved by both direct molecular evidence represented by NG (the number of shared genes) and systems-level factors such as common molecular mechanisms represented by the XD score.

Next, we compared the correlation of the XD scores and NGs obtained from disease pairs of the above six categories with RR and PHI scores. In terms of the XD score correlation (the second column in Table 1), the first three categories (+XDand+NG,+XD,and+NG) had high correlation coefficients both with RR and PHI scores with significant p-values from permutation test (see Methods). When neither +XD (positive XD score) nor +NG (at least one shared gene) criteria were applied (+NGnot+XDand+XDnot+NG), the correlation coefficients dropped significantly. In terms of the NG value (the third column in Table 1), no significant correlation was found in any disease pairs of the five categories.

In conclusion, these results indicate that the NG value or the XD score alone may not be a good indicator to explain disease comorbidity. In addition, when both of them were utilized to filter comorbid disease pairs, more strong correlation with clinical comorbidity score such as RR/PHI values was observed. This strongly supports that the combination of the two quantities (the XD score and the NG value) is a better prediction for comorbid disease pairs. We repeated the above analyses using the BioGRID network database^20^, which is smaller than the STRING database yet constructed by comprehensive curation, and obtained similar overall patterns (Supplementary Fig. 1 and Supplementary Table 2).

### Predicted disease network constructed based on shared genetic interaction

**Figure 3:**
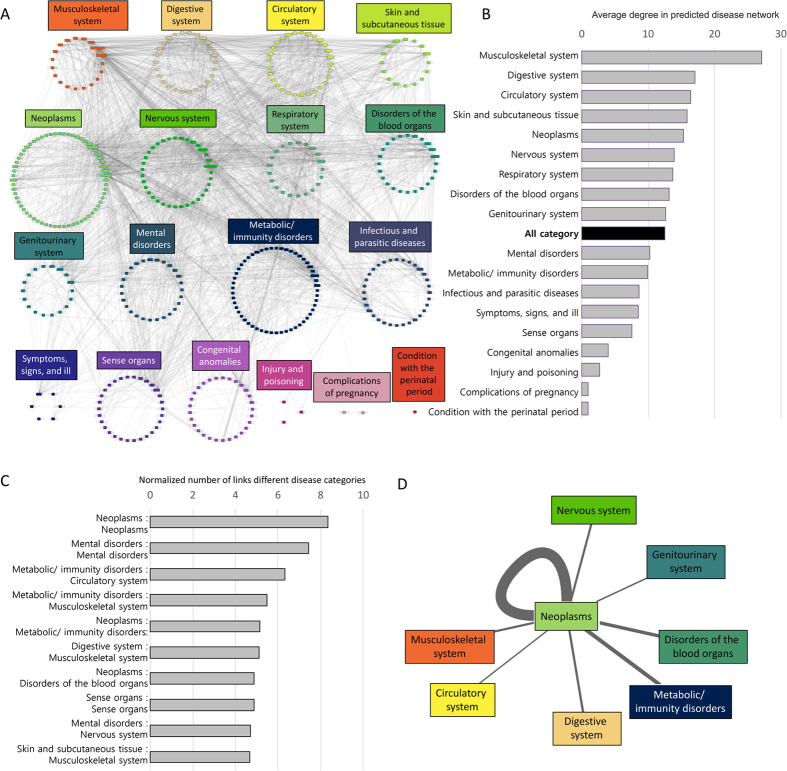
Construction of the predicted disease network based on XD score. (**A**) The top 10% of disease pairs having the highest XD score. The color of nodes indicates a disease category based on ICD-9 classification (Supplementary Table 3). (**B**) Average degree (number of links with other diseases) of diseases in the disease category. The average degree of all diseases in the disease network is 12.543 (marked as a black bar). Musculoskeletal system had the highest average degree, indicating that musculoskeletal system related diseases often accompany other diseases as complications or are frequently accompanied by other diseases as complications. (**C**) Top-ranked disease category pairs based on the normalized number of links between disease categories (including links between different disease category pairs as well as links within one disease category). Note that diseases in the neoplasm category have the highest intra-comorbidity patterns. (**D**) Illustration of distinct comorbidity patterns around the neoplasm category. The edge thickness represents the relative degree of the XD score between two disease categories.

A disease network that is constructed based on the XD score and NG score can provide the clue for etiology of comorbid diseases through the shared molecular origins such as common genes, shared interactions, or common biological pathways, which could not be explained in a comorbidity network constructed based on the RR/PHI values from clinical data. Thus, in this study, a disease comorbidity network (Fig. 3) was constructed from disease pairs having both the positive XD score and at least one shared gene, including 3,213 (3%) disease pairs out of total 97,665 ICD-9-CM disease pairs covering a total of 520 ICD-9-CM codes. The negative XD score indicates that the association of a disease pair is smaller than an average association level in a functional network. Therefore, we only considered disease pairs having the positive XD scores. We classified ICD-9-CM diagnosis codes into 18 pre-defined disease categories (Supplementary Table 3) and used them to hierarchically organize the predicted disease network.

To evaluate the predicted disease association in terms of actual disease comorbidity, 1,000,000 randomized disease networks were generated by edge shuffling, and the significance of the number of links across disease pairs within the same disease category as well as between two different disease categories was examined (Supplementary Fig. 2). As shown, 71% of disease categories were classified as significant, while only 39% of different disease category pairs were observed to have the significant number of links. This demonstrates the significance of predicated disease associations as a good indicator of disease comorbidity patterns.

As shown in Fig. 3A, diseases classified as “Neoplasms”, “Metabolic/immunity disorders”, “Circulatory system”, and “Nervous system” had prevalent association patterns with other diseases by representing a large number of nodes in the predicted disease network. Diseases belonging to the “Musculoskeletal system” category especially showed high association with many other diseases (Fig. 3B), indicating that most musculoskeletal diseases often are associated with other diseases by sharing the common biological mechanisms. “Digestive system”, “Circulatory system”, and “Skin and subcutaneous tissue” also showed similar patterns.

To quantify the association between disease categories and disease association patterns, we measured the average number of disease links among different disease categories. This number was then normalized to the number of diseases in each disease category. Results for top 10% of disease category pairs having high association are shown in Fig. 3C and D. Neoplasm-related diseases had high association pattern within themselves. This could be due to the etiological common mechanism of tumors^21^,^22^ (Fig. 3C). The next top-ranked highly-associated disease categories were mental disorders themselves, metabolic/immunity disorders with circulatory system, metabolic/immunity disorders with musculoskeletal system, and neoplasms with metabolic/immunity disorders^23^,^24^,^25^,^26^. As shown in Fig. 3D, clear associations between neoplasms and other diseases were observed. These high association patterns surrounding neoplasms may indicate that the existing complications are associated with cancers and that tumor patients might have poor survival and difficult recovery^27^,^28^.

## Discussion

Despite the lack of clinical data or incomplete understanding of pathology for diseases, our approach successfully identified associated disease pairs and the shared pathological mechanisms. Our approach has two main advantages: (i) by integrating direct disease-gene overlap and indirect molecular interactions, clinically meaningful comorbid disease pairs can be identified, and (ii) further investigation of shared pathological mechanisms of comorbid diseases is possible.

In order to demonstrate the utility of our approach, we predicted associated disease pairs and examined how well they are correlated with known comorbid disease pairs. We found that disease pairs with at least one common gene and the positive XD score have the strongest correlation (Supplementary Fig. 3). In addition, among total 97,665 disease pairs used in our analysis, 91,072 pairs did not share any gene (NG = 0), yet more than 40% of them shared significant amount of GO terms (Fisher’s exact test with a p-value cutoff 0.05, Supplementary Fig. 3). When the XD score was applied to extract the disease pairs for additional filtering, the fraction of disease pairs sharing the significant number of GO terms was more increased. This demonstrates that even disease pairs with NG = 0 do share common biological processes or molecular functions, and such common mechanisms can be explained by the XD score that utilizes propagated genes in a network.

The promising example of our approach is the detailed explanation for common molecular pathology associated with diseases pairs. For example, “Depressive disorder” (i.e., ICD-9: 311) and “Irritable bowel syndrome” (i.e., ICD-9: 564.1) were identified as an associated disease pair (Supplementary Table 1). Although they only share one disease-associated gene, they had strong comorbidity (i.e., RR: top 2%, PHI: top 0.03%), indicating strong associations at molecular level. Indeed, our approach revealed that common mechanisms such as “GO: 0004993, serotonin receptor activity”, “GO: 0007202, activation of phospholipase C activity”, and “GO: 0008219, circadian rhythm” were associated with these comorbid diseases, explaining their common pathologies. In addition, “Diabetes mellitus” (i.e., ICD-9: 250) and “Ankylosing spondylitis and other inflammatory spondylopathies” (i.e., ICD-9: 720) also revealed high comorbidity^29^. These diseases have been reported to be highly co-occurred diseases in the Asian population^30^. However, the exact pathology underlying such comorbidity has not been reported yet. We found that they shared 40% of enriched GO terms including “GO: 0005141, interleukin-10 receptor binding”, “GO: 0050776, regulation of immune response”, and “GO: 0032868, response to insulin”. Interleukin-10 (IL-10) is known to be associated with ankylosing spondylitis^31^. Uncontrolled serum level of IL-10 is also closely related to diabetes^31^. These common biological functions could effectively explain the pathology of such comorbid diseases. In addition, “Unspecified myeloid leukemia” (i.e., ICD-9: 205.9) with “Other specified congenital anomalies of spinal cord” (i.e., ICD-9: 742.59), “Hypoglycemia” (i.e., ICD-9: 251.2) with “Essential hypertension” (i.e. ICD-9: 401.9), and “Anemia” (i.e. ICD-9: 285.9) with “Intermediate coronary syndrome” (i.e. ICD-9: 411.1) were also identified as associated disease pairs by sharing molecular functions to explain the common pathobiology (Supplementary Table 1).

Currently, a disease and its complications are usually handled based on their manifestations. However, if we understand the fact that such comorbid diseases might have co-occurred based on perturbation of shared pathological mechanisms at molecular level, the therapy for such comorbid diseases can be changed. Instead of treating these comorbid diseases independently, we need to identify perturbed biological mechanisms that cause such diseases so that we can develop novel strategy for drug delivery and targeting.

## Methods

### Disease databases

We compiled the following four disease databases: OMIM^32^ (Online Mendelian Inheritance in Man, October 2014 version), HPO^33^ (Human Phenotype Ontology, October 2014 version), GAD^34^ (Genetic Association Database, November 2013 version), and DO^35^ (Disease Ontology, October 2014 version). Since each disease database uses various sources including genomic data and literature data, we incorporated all four databases to extract extensive disease-gene associations. For example, the GAD database is collected genetic associations based on polymorphism data. The OMIM database is a well-known repository of disease-gene associations based on genetic information mostly limited to Mendelian disorders. Data in the HPO database are collected and annotated through medical literature and various experiment data. The DO database represents a comprehensive knowledge base of 8043 inherited developmental human diseases. It provides extensive cross-mapping with MeSH (Medical Subject Headings), ICD (International Classification of Diseases), and OMIM identifiers. Diseases and genes in different databases are annotated with different identifiers. Since there is disease-identifier inconsistency among heterogeneous disease databases, an integration process for disease name normalization (Supplementary Fig. 4) was applied, covering a total of 1,439 associations among 1,022 diseases and 4,914 genes. The integration process is done though OMIM_ID. As shown in Supplementary Fig. 4, the HPO database provided the mapping information between HPO_ID and OMIM_ID. The DO database also provided the mapping information between DO_ID and OMIM_ID, and the GAD database also had mapping information between GAD_ID and OMIM_ID. Then, the mappings of OMIM_ID and ICD-9-CM codes were obtained from two sources. One was from previous studies^11^,^12^ mapped through manual curation. The other was from the DO database providing mapping between DO_ID and ICD-9-CM through OMIM_IDs.

### US Medicare Data

The US Medicare is a national social insurance program, administered by the US federal government. It provides health insurance for age 65 and older people and maintains all history of health records for approximately 40 million people. In our study, we analyzed the US Medicare data of approximately 13,038,014 individuals, who had the 32,341,347 inpatient hospital visits^15^. This data includes all diagnosis terms (e.g. ICD-9) which were clinically assigned to each of the patients.

### Network databases

The STRING 9.1 network database^16^, one of the largest databases of direct protein-protein interactions and indirect functional interactions constructed from various data sources, was used. It contained 20,772 proteins with Ensembl protein identifiers with 2,425,315 interactions among them. Because our gene sets were represented by Entrez identifiers, Ensembl protein identifiers in the original STRING database were converted to Entrez identifiers by using mapping information in the STRING database. This resulted in 18,074 genes with 2,153,757 interactions among them.

### Comorbidity score of a pair of diseases

Relative Risk (RR) and Phi-correlation (PHI) have been popularly used^7^,^12^,^15^,^19^ to reflect the proportion of the number of patients that actually share diseases. We denoted that *C*_*ij*_ was the number of patients who were diagnosed with both diseases *i* and *j*. The numbers of patients having disease *i* and *j* were *I*_*i*_ and *I*_*j*_, respectively. *N* was the total number of patients. The relative risk of two diseases *i* and *j* were given by *RP*_*ij*_/*IP*_*ij*_, where *RP*_*ij*_ was the co-occurrence probability (i.e. *C*_*ij*_/*N*) of disease *i* and disease *j. IP*_*ij*_ was the joint probability of each of two diseases assuming they were independent (i.e. (*I*_*i*_/*N*)*(*I*_*j*_/*N*)). PHI, Pearson’s correlation for binary variables, was defined as 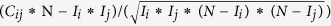. To quantitatively represent comorbid tendency between two diseases (d_1_, d_2_) in our study, we adapted the XD score measure^18^ to represent the functional closeness of two disease-associated gene sets. The XD score was calculated as follows. In the first step, a score vector of a specific disease (d_1_) was created by setting 1 for all associated genes and 0 for all others. In the second step, the score vector was iteratively updated based on Random Walk with Restart (RWR) algorithm with a restart probability of p = 0.9 by using the STRING network database. In the third step, the XD score was calculated by using the updated score vector and associated genes of the other disease (d_2_). Genes in the updated score vector were sorted in descending order based on their association scores and discretized into equal-sized bins of the scores. The size and range of the bins were defined by the following equations:


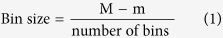



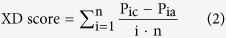



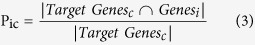



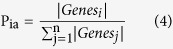


where *TargetGenes*_*c*_ and *Genes*_*i*_ represented the set of genes associated with the current target disease (d_2_) and the set of genes in the i^th^ bin respectively. *P*_*ic*_ was the fraction of genes associated with the *current* target disease (d_2_) in the i^th^ bin. *P*_*ia*_ was the fraction of *all* genes in the i^th^ bin. *M* and *m* were the maximum and minimum scores of the updated score vector respectively, *n* was the number of bins (in our study, *n* = 10 is used), and *i* was the current bin number. The reverse case (d_2_ was used for RWR) was also considered. The XD score was calculated using the same equations. The final XD score was defined as the minimum value of the two scores from d_1_ and d_2_ as the start of the RWR algorithm in order to represent reliable relatedness.

### Estimating significance of correlation

We assessed the significance of observed correlation coefficient by comparing it to the set of correlation coefficients obtained from randomly permuted gene sets. The p-value for the Pearson’s correlation coefficient (PCC) between genetic variables including the XD score and comorbid tendency scores (i.e., RR and PHI-correlation) in Table 1 was estimated using the Monte Carlo sampling methods. We repeatedly permuted the values in the lists of each two variables and calculated PCCs. This was performed two million times to obtain the distribution of PCCs. The p-value was the fraction of total PCCs, which is larger than our correlation coefficient.

### GO enrichment test for associated disease pairs

Gene Ontology (GO) enrichment analysis was performed with each disease associated gene sets. GO terms enriched with one disease were identified with a hypergeometric test between a disease-associated gene set and GO-annotated gene sets with cutoff p-value of 0.05. After obtaining enriched GO terms for each disease, common GO terms were identified for each of comorbid disease pairs. They were used to explain common pathology for these comorbid diseases.

## Additional Information

**How to cite this article**: Ko, Y. *et al*. Identification of disease comorbidity through hidden molecular mechanisms. *Sci. Rep.* **6**, 39433; doi: 10.1038/srep39433 (2016).

**Publisher's note:** Springer Nature remains neutral with regard to jurisdictional claims in published maps and institutional affiliations.

## Supplementary Material

Supplementary Information

Supplementary Table 1

Supplementary Table 2

Supplementary Table 3

## Figures and Tables

**Table 1:** Correlation between different measures of disease comorbidity.

Criteria for disease pairs	XD		NG	
	RR	PHI	RR	PHI
+XDand+NG	0.2640 (6.59 × 10^−4^)	0.1377 (2.687 × 10^−3^)	−0.0065 (0.3789)	0.0251 (0.1329)
+XD	0.2592 (5.20 × 10^−4^)	0.1432 (2.29 × 10^−3^)	−0.0047 (0.2466)	0.0344 (0.0474)
+NG	0.2407 (3.26 × 10^−4^)	0.1267 (1.184 × 10^−3^)	−0.0023 (0.1753)	0.0360 (0.0338)
+NGnot+XD	0.0059 (0.0764)	−0.0209 (0.9999)	0.0096 (0.0892)	0.0288 (0.0357)
+XDnot+NG	0.0886 (0.0132)	0.0723 (0.0305)	NA	NA
not+XDnot+NG	−0.0029 (0.8218)	0.0040 (0.1130)	NA	NA
ALL	0.1557 (6.67 × 10^−6^)	0.0759 (0)	0.0013 (0.0521)	0.0254 (0.0016)

Numbers represent Pearson’s correlation coefficients for XD scores against RR/PHI scores or for NG values against RR/PHI scores calculated from different sets of disease pairs constructed by different categories shown at the first column; p-values in parenthesis are from permutation tests.

+XD: disease pairs having positive XD scores.

+NG: disease pairs having at least one common gene.

+XDand +NG: disease pairs having both positive XD score and at least one common gene.

+NGnot+XD: disease pairs having at least one common gene but without having positive XD scores.

+XDnot+NG: disease pairs having positive XD scores but without having common disease genes.

not+XDnot+NG: disease pairs having negative XD scores and without having common disease genes.
