# Supplementary material for: Identification of disease comorbidity through hidden molecular mechanisms
Source: Sci Rep. 2016 Dec 19;6:39433. doi: 10.1038/srep39433 (PMC5172201; doi:10.1038/srep39433)
Supplement: Supplementary Information [file srep39433-s1.pdf]

Supplementary Information

Identification of disease comorbidity through hidden molecular mechanisms

Younhee Ko[1], Minah Cho[2], Jin-Sung Lee[1], and Jaebum Kim[2,*]

[1]Department of Clinical Genetics, Department of Pediatrics, Yonsei University College of Medicine, Seoul 03722, South Korea
[2]Department of Stem Cell and Regenerative Biology, Konkuk University, Seoul 05029, South Korea
* jbkim@konkuk.ac.kr

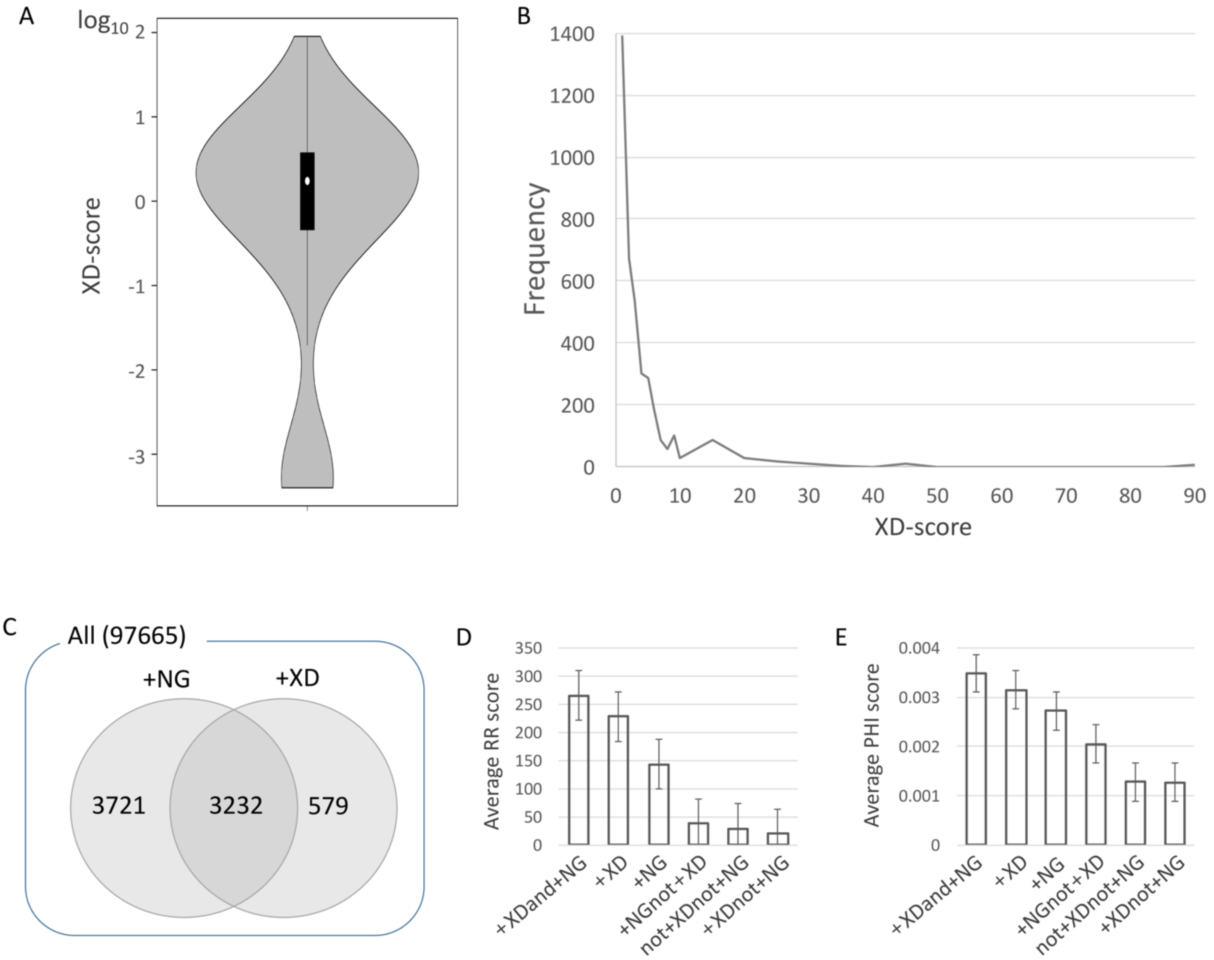

Supplementary Figure 1. Statistics of comorbidity measures for disease pairs in the US Medicare data based on the BioGRID database. (A) The distribution of the log-scaled XD scores. (B) The distribution of the positive XD scores. (C) The numbers of disease pairs chosen by different quantities (+NG: disease pairs having at least one common gene, +XD: disease pairs having the positive XD scores). (D) The average and standard errors of RR scores of disease pairs chosen by different quantities. (E) The average and standard errors of PHI scores of disease pairs chosen by different quantities. (+XDand+NG: disease pairs having both the positive XD scores and at least one common gene, +NGnot+XD: disease pairs having at least one common gene but without the positive XD scores, +XDnot+NG: disease pairs having the positive XD scores but without sharing genes, and not+XDnot+NG: disease pairs having the negative or zero XD scores without sharing any gene).

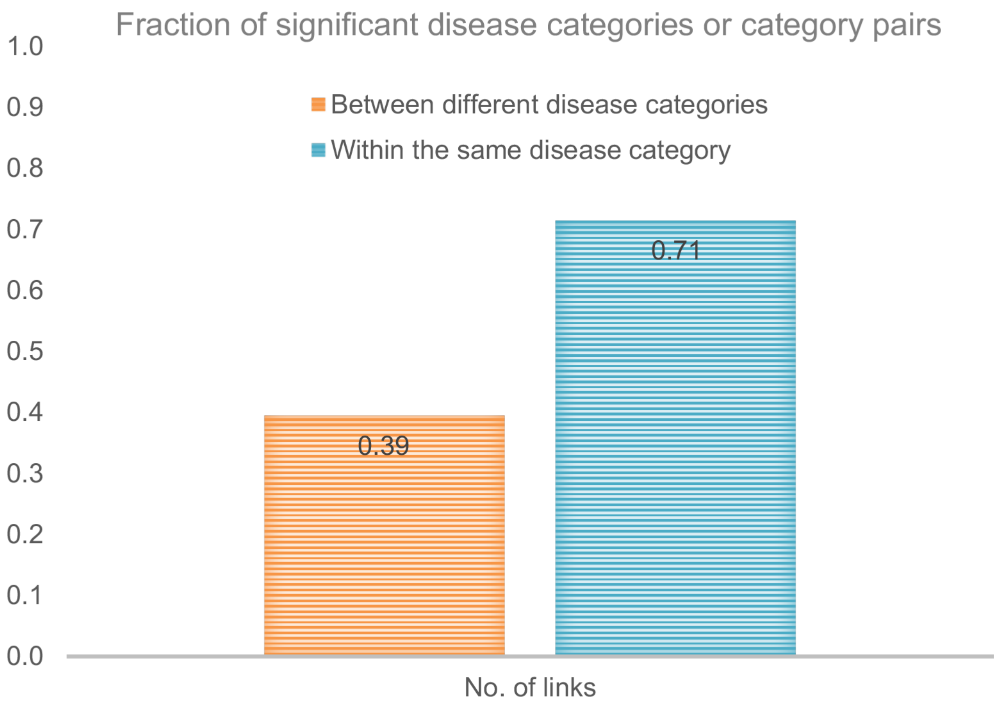

Supplementary Figure 2. Fraction of significant disease categories or category pairs. 1,000,000 randomized disease networks were made by edge shuffling and used to measure the significance of predicted disease categories or category pairs. The statistical significance was measured in terms of the number of links among diseases within the same disease category (the "Within the same disease category" legend) as well as between different disease categories (the "Between different disease categories" legend) with p-value cutoff 0.05.

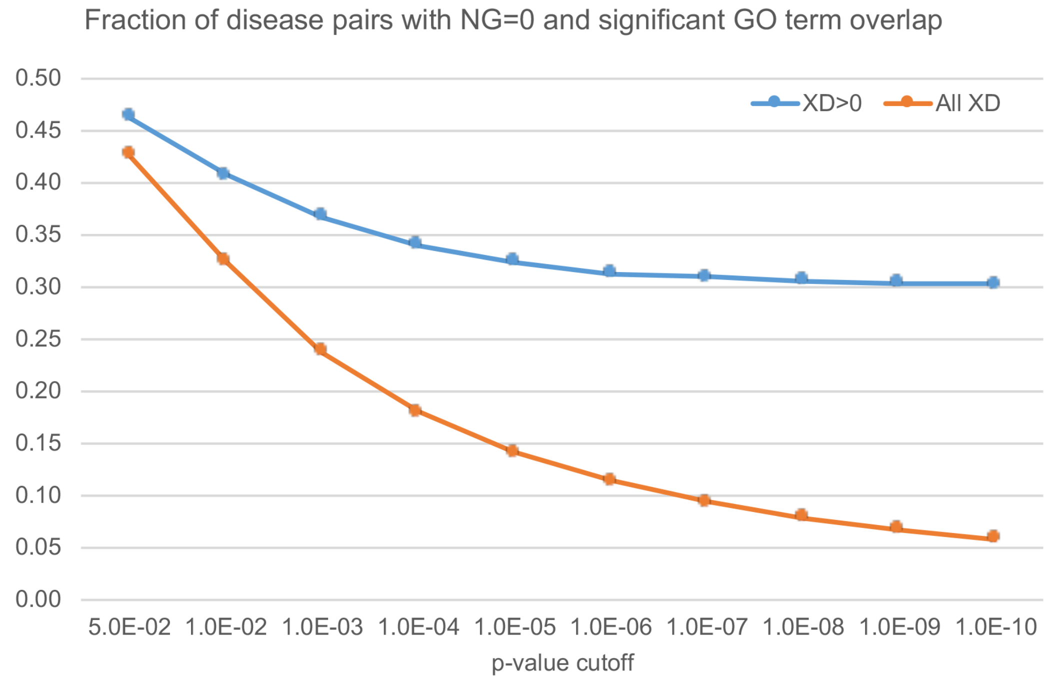

Supplementary Figure 3. Fraction of disease pairs with NG=0 that have significant GO term overlap. Among total 97,665 disease pairs, 91,072 pairs without shared disease-associated genes (NG=0) were collected, and the significance of GO term overlap was measured using the Fisher's exact test. X-axis represents various cutoff p-values used to determine the significance and Y-axis shows the fraction of significant disease pairs. The orange line indicates the result from 91,072 pairs. Similar test was also conducted for the subset of disease pairs with the positive XD score (total 796) and it is shown as a blue line.

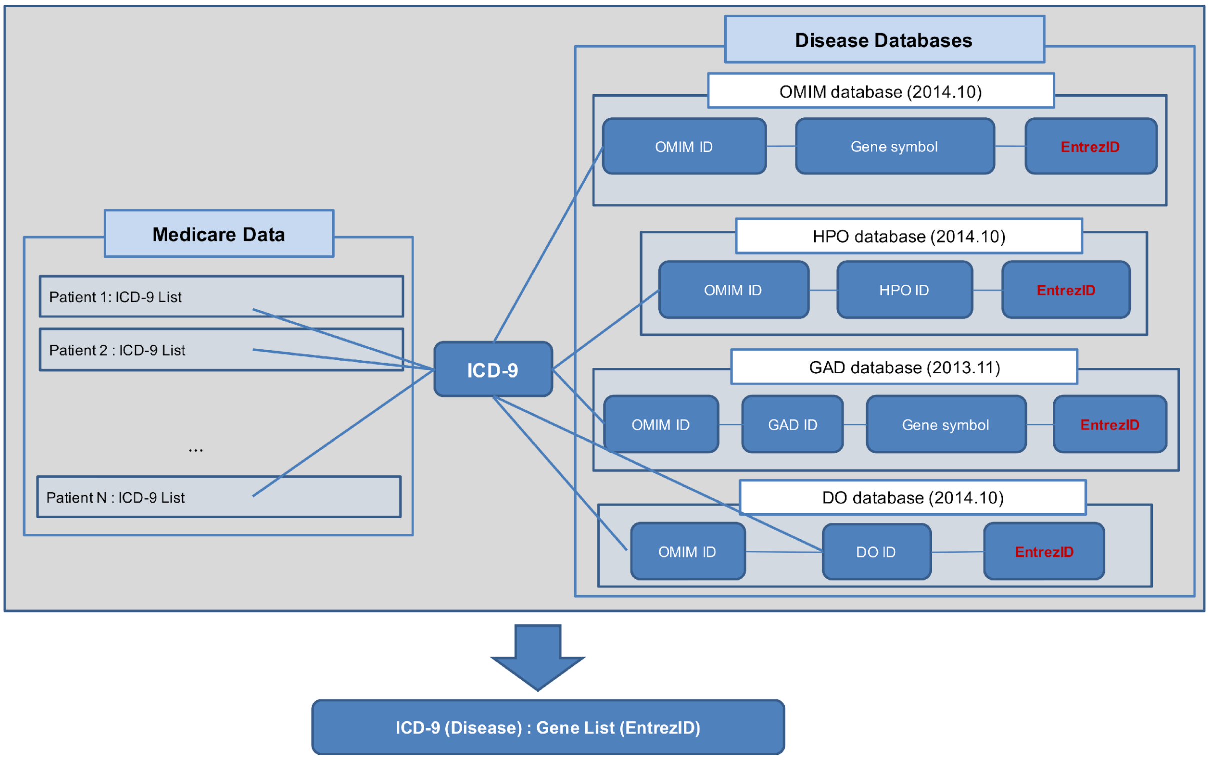

Supplementary Figure 4. Integration process for resolving disease-identifier inconsistency among heterogeneous disease databases.
